# Ebola Hemorrhagic Fever Associated with Novel Virus Strain, Uganda, 2007–2008

**DOI:** 10.3201/eid1607.091525

**Published:** 2010-07

**Authors:** Joseph F. Wamala, Luswa Lukwago, Mugagga Malimbo, Patrick Nguku, Zabulon Yoti, Monica Musenero, Jackson Amone, William Mbabazi, Miriam Nanyunja, Sam Zaramba, Alex Opio, Julius J. Lutwama, Ambrose O. Talisuna, Sam I. Okware

**Affiliations:** Author affiliations: Ministry of Heath, Kampala, Uganda (J.F. Wamala, L. Lukwago, M. Malimbo, M. Musenero, J. Amone, S. Zaramba, A. Opio, J.J. Lutwama, A.O. Talisuna, S.I. Okware);; World Health Organization Country Office, Kampala (Z. Yoti, W. Mbabazi, M. Nanyunja);; African Field Epidemiology Network Secretariat, Kampala (P. Nguku)

**Keywords:** Ebola virus, Ebola hemorrhagic fever, hemorrhagic fever, filovirus, viruses, research

## Abstract

During August 2007–February 2008, the novel *Bundibugyo ebolavirus* species was identified during an outbreak of Ebola viral hemorrhagic fever in Bundibugyo district, western Uganda. To characterize the outbreak as a requisite for determining response, we instituted a case-series investigation. We identified 192 suspected cases, of which 42 (22%) were laboratory positive for the novel species; 74 (38%) were probable, and 77 (40%) were negative. Laboratory confirmation lagged behind outbreak verification by 3 months. *Bundibugyo ebolavirus* was less fatal (case-fatality rate 34%) than Ebola viruses that had caused previous outbreaks in the region, and most transmission was associated with handling of dead persons without appropriate protection (adjusted odds ratio 3.83, 95% confidence interval 1.78–8.23). Our study highlights the need for maintaining a high index of suspicion for viral hemorrhagic fevers among healthcare workers, building local capacity for laboratory confirmation of viral hemorrhagic fevers, and institutionalizing standard precautions.

## Abstract 2

Case-fatality rate is lower for this strain than for the other 3 Ebola species known to be pathogenic to humans.

Ebola hemorrhagic fever (EHF) is a severe, often fatal disease of humans and nonhuman primates caused by a single-stranded RNA virus belonging to the *Filoviridae* family. The virus was first isolated in 1976 after hemorrhagic fever outbreaks in Zaire and Sudan that resulted in >250 deaths (*1**,**2*). Before the outbreak described here, 4 distinct species of Ebola virus were known: *Zaire ebolavirus*, *Sudan ebolavirus*, *Côte d’Ivoire ebolavirus*, and *Reston ebolavirus*.

Although the reservoirs for the virus and mechanisms of transmission have not been fully elucidated, a recent study reported that 4% of bats from Gabon were positive for Zaire Ebola virus immunoglobulin (Ig) G (*3*). Initial human infection presumably occurs when humans are exposed to infected body fluids of the animal reservoir or intermediate host. Thereafter, person-to-person transmission occurs through direct contact with body fluids of an infected person (*1**,**2**,**4*). After an incubation period of 1–21 days (*1**,**2**,**5*), an acute febrile illness develops in infected persons; the disease is characterized by the sudden onset of fever, chills, headache, and myalgia, followed later by rash, sore throat, nausea, vomiting, diarrhea, and abdominal pain (*1**,**2**,**5*). Approximately half of infected persons manifest hemorrhagic signs, e.g., bleeding from the nasal cavity, passing of blood in the urine, and/or gastrointestinal and vaginal bleeding (*5**,**6*). The case-fatality rate (CFR) for Zaire Ebola virus and Sudan Ebola virus (SEBOV) infections varies from 53% to 90% (*7*). The first EHF outbreak in Uganda occurred in 2000 and affected Gulu, Masindi, and Mbarara districts, with a total of 425 case-patients and 224 deaths (CFR 53%). The outbreak was caused by the *Sudan ebolavirus* species (*8*).

On November 5, 2007, the Uganda Ministry of Health (UMOH) received a report of the deaths of 20 persons in Bundibugyo district, western Uganda. However, UMOH had received initial reports of 2 suspected cases of a febrile diarrheal illness on August 2, 2007. A UMOH team investigated these 2 cases, but the findings were not conclusive because of inadequate in-country laboratory capacity. In the second report, ill persons had an acute febrile hemorrhagic illness, and tests conducted by the US Centers for Disease Control and Prevention (Atlanta, GA, USA) confirmed the illness as EHF on November 29, 2007.

## Methods

### Outbreak Site

**Figure 1:**
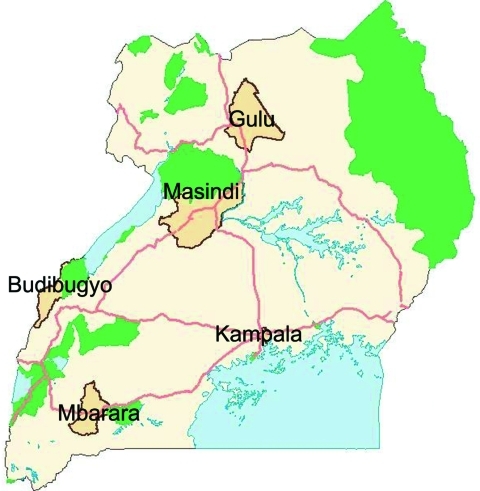
Major towns in Uganda. Districts and major towns share the same names. Green shading, national parks; red lines, main roads; blue shading, perennial lakes.

Bundibugyo district is located in western Uganda (Figure 1). Approximately 60% of the district is covered by the Rwenzori Mountains and Semliki National Park and Game Reserve, which have a broad range of wildlife, including primates. The district has a population of 253,493 and a population density of 108 persons/km^2^ (*9*), 1 hospital, and 26 health centers. The main economic activities are cocoa farming, fishing, and tourism. Hunting of wild animals is common among settlements near the national park.

### Epidemiologic Activities

During November 29, 2007–February 20, 2008, village health teams conducted active searches in the communities through daily door-to-door visits in their respective villages while investigation teams of local and international experts reviewed case notes at health facilities and verified suspected cases reported by the village health teams. Cases were categorized as suspected EHF, probable EHF, and confirmed EHF (Table 1).The UMOH developed working case definitions and established an enhanced surveillance system for identifying suspected case-patients in the affected district and in contiguous districts.

**Table 1:** Case definitions for epidemiologic investigation of EHF outbreak, Bundibugyo district, Uganda, 2007–2008*

Classification	Definition
Suspected case	Sudden onset of fever and at least 4 of the following symptoms in a resident of or visitor to the affected subcounties in Bundibugyo district: vomiting, diarrhea, abdominal pain, conjunctivitis, skin rash, unexplained bleeding from any body part, muscle pain, intense fatigue, difficulty swallowing, difficulty breathing, hiccups, or headache since August 1, 2007, OR sudden onset of fever in any person who had had contact with a person with suspected, probable, or confirmed EHF, OR sudden death in a person in the community without any other explanation.
Probable case	Suspected EHF in any person (dead or alive) with at least 3 of the following symptoms; vomiting, diarrhea, or unexplained bleeding from any site, conjunctivitis, or skin rash; AND with an epidemiologic link to a person with probable or confirmed EHF, OR either no specimen collected for laboratory testing or a negative laboratory result in a specimen collected 0–3 days after onset of symptoms in a person with suspected EHF.
Confirmed case	Laboratory confirmation of infection by isolation of virus from any body fluid or tissue, OR detection of viral antigen in any body fluid or tissue by antigen-detection ELISA, reverse-transcription–PCR, or immunohistochemistry, OR demonstration of serum Ebola virus–specific IgG antibodies by ELISA, with or without IgM, in any person with suspected or probable EHF.
Contact	A person who had slept in the same household and/or had direct physical contact with a person (dead or alive) with suspected, probable, or confirmed EHF and/or had been exposed to an infected person or to an infected person’s secretions, excretions, tissues, or linens within 3 weeks after that person’s onset of illness.

*EHF, Ebola hemorrhagic fever; Ig, immunoglobulin.

### Investigative Activities

Investigation teams actively searched for case-patients in health facilities and communities and retrospectively reviewed hospital records. Increased awareness of the disease through public education campaigns and the media facilitated reporting of suspected cases to health authorities. Village health teams assisted in case identification and reports and contact follow-up at the community level. Clinical and epidemiologic data were systematically collected from persons with suspected, probable, and confirmed cases. Proxies (usually parents, spouses, or adult siblings) were interviewed for information about case-patients who had died before an interview could be conducted. Persons with suspected cases identified in the community were transported by a mobile ambulatory team to designated isolation facilities. A triage desk was established in the outpatient departments of each health facility in the district to screen for suspected cases and notify the surveillance system. Clinical specimens, including blood, were collected for laboratory testing from all persons with suspected EHF. All contacts were entered into, and follow-up schedules were drawn by using, the Field Information Management System database (*10*).

### Laboratory Methods

Five milliliters of blood was collected from all persons with suspected EHF at least 4 days after symptom onset when possible. Specimens initially were transported to the Uganda Virus Research Institute (UVRI) and then transferred to US Centers for Disease Control and Prevention for laboratory analysis. However, on December 4, a laboratory was set up at UVRI, and subsequent specimens were tested there, as described by Towner et al. (*11*). Virus isolation was not attempted at UVRI.

### Data Analysis

We used Epi Info software version 3.4 (*12*) to create a database into which information from individual case investigation forms was entered and updated daily. The age and sex population structure and projections for Bundibugyo district were obtained from the population and housing census 2002 data (*9*), and attack rates were computed by using the district population projections. Current geographic maps were obtained from the World Health Organization Health Mapper Mapping Software, version 4.2 (*13*).

We tabulated risk factors by case status and calculated odds ratios (ORs) using as the reference group persons with suspected EHF who had negative test results. To control for confounding and to test for effect modification, we entered variables with p values <0.1 into a multivariable logistic regression model. By using backward elimination, we eliminated all variables that were not statistically significant at p<0.05. The model had the following co-variables: admission to hospital or visit with a sick person, consultation with traditional healer, participation in funeral rituals, travel to area with cases, contact with a known suspected/confirmed case-patient, contact with wildlife, subcounty of residence, age, and sex. The Uganda National Council of Science and Technology expedited ethical review and clearance because the data were being collected to guide outbreak control.

## Results

### Outbreak Response

UMOH declared an EHF outbreak on November 29. Consequently, the National Task Force, composed of both local and international partners, was activated to determine the magnitude of the outbreak and coordinate immediate outbreak response.

All case-patients were treated in isolation wards set up in Kampala at the National Referral Hospital, Mulago, and in Bundibugyo at Kikyo and Bundibugyo health facilities. Health workers on the case-management teams had participated in patient management during the SEBOV outbreak in Gulu in 2000, thus, their experience provided a valuable resource during the response. Countrywide social mobilization and health education during the outbreak involved developing and disseminating health education materials and messages through mobile video shows, community meetings with opinion leaders, radio talk shows, and newspaper inserts.

### Index Case Investigation

The putative index patient was a 26-year-old woman from Kabango village, Kasitu subcounty, in Bundibugyo district. Hunting spears were found at her home, but hunting as a practice was denied. Fever and general weakness developed in this woman, for which she was hospitalized on August 1. She delivered a preterm infant the following day. Diarrhea and difficulty breathing developed, but hemorrhagic manifestations did not appear. She died on August 4.

In this cluster, 9 case-patients and 6 deaths (the neonate, sister, mother, and 2 nieces of the index patient) (CFR 67%) were reported. The mother and sister were involved in nursing and handling the remains of the index patient because, at the time, barrier nursing and supervised burials had not been initiated. Because these persons were affected before the outbreak was confirmed, the high CFR was attributable to inadequate supportive care. Three survivors were tested; 2 were positive for *Bundibugyo ebolavirus*–specific IgG.

### Description of Cases

Illnesses in 192 persons met the definition for suspected EHF. Of these, 42 (22%) were laboratory confirmed as positive for a novel Ebola virus species *(Bundibugyo ebolavirus*); 74 (38%) remained probable, and 76 (40%) were laboratory negative and classified as noncases.

Overall, 39 of the 116 persons with confirmed and probable EHF died (CFR 34%). For male patients, the CFR was 35% (23/65) and for females, 31% (16/51). The CFR for confirmed EHF was 33% (14/42) and for suspected EHF, 34% (25/74). Case-patients’ ages ranged from 3 weeks to 70 years (mean 34 years, median 35 years). Most 40 (36%) case-patients were crop farmers; 14 (12%) were healthcare workers.

### Symptoms

The median incubation period from contact with an infected person to symptom onset was 7 days (range 2–20 days). The most frequent symptoms were fever (100%), intense fatigue (92%), headache (87%), abdominal pain (87%), vomiting (83%), and diarrhea (83%). Bleeding manifestations (including hematuria; hematemesis; bleeding from the eyes, nose, and vagina; and/or bloody stool) occurred in 52% of case-patients, and most (59%) case-patients who died had some form of bleeding. Other symptoms reported were dysphagia (54% of case-patients), dyspnea (52%), and rash (50%). Median duration from symptom onset to recovery was 10 days (range 2–26 days). Median duration from symptom onset to death was 10 days (range 3–21 days).

**Figure 2:**
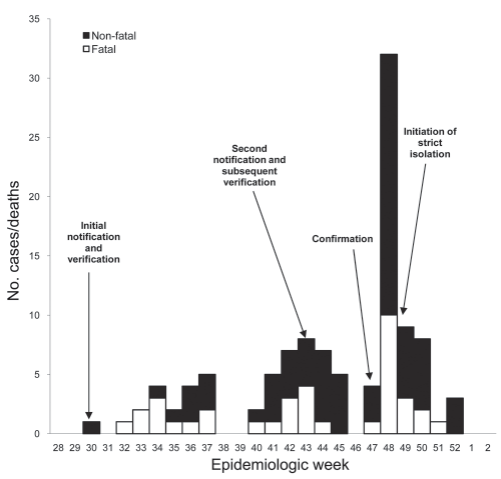
Ebola outbreak, by week of onset for probable and confirmed cases (n = 116), Bundibugyo district, Uganda, August–December 2007.

The outbreak showed 3 successive cycles of transmission (Figure 2). Each transmission cycle lasted 6 weeks, with an interval of 3–11 days, and was characterized by a typical epidemic peak followed by gradual decline. The highest peak occurred on November 25. Despite the timely detection and initiation of investigations during the 30th epidemiologic week, the successive transmission cycles indicated delayed laboratory confirmation and declaration of the outbreak. Outbreak confirmation was delayed until the 48th epidemiologic week, resulting in inadequate control measures before the outbreak was confirmed.

### Geographic Characteristics and Risk Factors

The outbreak was confined to Bundibugyo district. More than 97% of cases were detected in 4 of the 10 subcounties in the district (Kasitu, Bundibugyo town council, Bubukwanga, and Busaru), with >54% of cases occurring in Kasitu subcounty (Table 2).

**Table 2:** Geographic distribution of persons with Ebola hemorrhagic fever, Bundibugyo district, Uganda, 2007–2008

Subcounty	Population	No. cases	No. deaths	Case-fatality rate, %	Attack rate*
Kasitu	33,968	63	18	29	185
Bundibungyo town council	17,590	25	8	32	142
Bubukwanga	23,398	17	7	41	73
Busaru	40,547	8	3	38	20
Harugali	29,162	1	1	100	3
Karugutu	19,384	1	1	100	5
Bubandi	22,063	1	1	100	5
Other subcounties	81,879	0	0	0	0
Total	267,991	116	39	34	43

*Per 100,000 population.

The overall attack rate in the district was 43 cases/100,000 population. The highest rate occurred in Kasitu subcounty, followed by the rate in Bundibugyo town council (within Bundibugyo district (Table 2). By sex, the attack rate for men was higher than that for women (64 vs. 47/100,000 population). By age group, the attack rate was higher for persons 41–50 years of age than for persons 51–60 years of age (146 vs. 122/100,000 population).

### Analysis of Possible Risk Factors

All case-patients were investigated for exposures within 3 weeks before development of symptoms. To better determine risk factors for the disease, we conducted a bivariate analysis using the 76 non–case-patients as the reference group to separately assess persons with probable and confirmed EHF (Table 3). Before the institution of strict isolation policies, visitors had direct contact with patients through shaking of hands, hugging, or contact with potentially infected surfaces. Patients with confirmed EHF (OR 8.71, 95% confidence interval [CI] 3.03–26.30) and patients with probable and confirmed cases combined (OR 2.56, 95% CI 1.35–4.85) were significantly more likely to have visited sick persons or to have visited the hospital 3 weeks before becoming sick. Consultation with a traditional healer within 3 weeks before illness onset was not significantly associated with having EHF (OR 0.16, 95% CI 0.01–1.15) (Table 3).

**Table 3:** Bivariate analysis of risk factors for Ebola viral hemorrhagic fever, Bundibugyo district, Uganda, 2007–2008*

Potential risk factor	Probable case, n = 74	Confirmed case, n = 42	Probable/confirmed case, n = 116	Noncase, n = 76 (ref.)
Hospitalized/visited hospital, no. (%)	38 (51.40)	36 (85.70)	74 (63.79)	31 (40.80)
OR (95% CI)	1.5 (0.8–3.1)	**8.7 (3.0–26.3)**	**2.6 (1.4–4.9)**	1
p value	0.2	**<0.001**	**<0.05**	
Consulted traditional healer, no. (%)	1 (1.4)	0	1 (0.9)	4 (5.3)
OR (95% CI)	0.25 (0.01–2.4)	Undefined	**0.2 (0.01–1.5)**	1
p value	0.2		**0.06**	
Participated in funeral rituals, no. (%)	43 (58.1)	32 (76.2)	75 (64.7)	23 (30.2)
OR (95% CI)	**3.2 (1.6–6.6)**	**7.4 (2.9–19.3)**	**4.22 (2.2–8.2)**	1
p value	**<0.001**	**<0.001**	**<0.001**	
Traveled before illness, no. (%)	**29 (39.20)**	11 (26.20)	**40 (34.50)**	15 (19.74)
OR (95% CI)	**2.62 (1.2–5.8)**	1.4 (0.5–3.8)	**2.1 (1.0–4.5)**	1
p value	**<0.05**		**0.03**	
Had contact with person with known suspected case, no. (%)	48 (64.90)	42 (100.00)	90 (77.60)	43 (56.58)
OR (95% CI)	1.4 (0.7–2.9)	Undefined	**2.7 (1.35–5.24)**	1
p value	0.3		<0.05	
Had contact with wildlife, no. (%)	1 (1.4)	0	1 (0.9)	1 (1.3)
OR (95% CI)	1.0 (0.0–38.4)	Undefined	0.7 (0.02–24.30)	1
p value	1.0		0.8	
Male sex, no. (%)	40 (54.00)	25 (59.52)	65 (56.00)	37 (29.31)
OR (95% CI)	1.2 (0.6–2.5)	1.6 (0.7–3.6)	1.3 (0.7–2.5)	1
p value	0.5	0.3	0.3	
Age 41–60 y, no. (%)	18 (24.30)	16 (38.10)	34 (0.90)	18 (1.32)
OR (95% CI)	1.0 (0.5–2.3)	2.0 (0.8–4.9)	1.3 (0.7–2.7)	1
p value	0.9	0.1	0.4	

*Ref., reference; OR, odds ratio; CI, confidence interval. **Boldface** indicates significant associations.

The other risk factor identified was participation in funeral rituals before onset of illness. The funeral rituals, performed by close relatives, involved washing and dressing the body of the decedent. Patients with probable EHF (OR 3.20, 95% CI 1.55–6.64) and confirmed EHF (OR 7.37, 95% CI 2.89–19.27) were significantly more likely than persons in the reference group to have participated in funeral rituals before they became sick (Table 3). Similarly, when both groups of patients with probable and confirmed EHF were combined (OR 4.22, 95% CI 2.17–8.24), the members of the new group were significantly more likely to have participated in funeral rituals before becoming sick (Table 3).

Ebola case-patients were asked whether they had had any form of contact with a person known to have suspected or confirmed EHF. Patients with probable and confirmed EHF combined were significantly more likely (OR 2.66, 95% CI 1.35–5.24) to have had contact with a person known to have suspected or confirmed EHF before becoming sick (Table 3).

We performed multivariate analysis using binary logistic regression to control for confounding and to test for effect modification. After backward elimination, participating in funeral rituals remained as the sole significant risk factor associated with being a probable/confirmed case-patient (adjusted OR 3.83, 95% CI 1.78–8.23).

## Discussion

The EHF epidemic in Bundibugyo district in western Uganda during August 1, 2007–February 20, 2008, was caused by a new species of the virus, *Bundibugyo ebolavirus*. It most likely was transmitted from wildlife located within Semliki National Park. The median incubation period of 1 week was less than that reported previously (*5*) but within the range reported in other Ebola outbreaks (*1**,**2*). In addition, patients’ symptoms were similar to those reported previously, except for the manifestation of bleeding, which occurred >2× more often than during the SEBOV outbreak in Gulu in 2000 (*5*).

The *Bundibugyo ebolavirus* outbreak (CFR 34%) caused a lower proportion of deaths than did the SEBOV outbreak in Gulu (CFR 53%) (*5*). Similarly, the CFR for the Bundibugyo outbreak was lower than that reported from other outbreaks outside Uganda (*1**,**2*), which indicates either that the new virus strain may be less virulent or that improved interventions led to more timely case identification and better case management.

During the Bundibugyo outbreak, vital functions were sustained by supportive treatment, including administering antipyretics, monitoring fluid balance, and giving antibacterial or antimalarial drugs for concurrent bacterial or protozoal infections. Oral rehydration and oral administration of antibacterial drugs were encouraged for all patients, provided they were conscious and not vomiting; otherwise, fluids and antibiotics were administered intravenously. To streamline case detection, village health teams and ambulance teams were trained early to conduct active case search and referral. Strict isolation measures included the establishment of triage in all health facilities, designation of isolation wards, training of healthcare workers in adherence to standard precautions, barrier nursing, supervised burial, and ambulance services.

During the Bundibugyo outbreak, case-patients were more likely than non–case-patients to have participated in funeral rituals. The practice exposes contacts to infectious body fluids that have been associated with acquiring EHF (*4*).

Fourteen health workers were infected during the Bundibugyo outbreak before strict isolation procedures were initiated. During the SEBOV outbreak in Gulu, 64% of health workers were infected after isolation wards were established (*14*).

The outbreak response had 2 challenges and at least 1 limitation. Investigations at the local and national levels were conducted in a timely manner, but the lack of capacity for laboratory confirmation delayed initial outbreak confirmation and therefore the initiation of an appropriate response. Because hunting in the national parks is illegal, attempts to link the Bundibugyo outbreak to wildlife were futile because none of the families investigated admitted to participating in hunting. The use of hospitalized patients as a comparison group was economical, but their illnesses could have been related to risk factors for *Bundibugyo ebolavirus* infection, hence rendering those risk factors undetectable.

We recommend that an index of suspicion for Ebola viruses (and Marburg virus) be maintained for clusters of cases with fever of sudden onset, intense fatigue, abdominal upsets, and evidence of person-to-person transmission. In concert with timely initiation of active case searching, use of ambulance and burial teams, and strict adherence to patient isolation practices, an index of suspicion should ensure mitigation of the identified risk factors.
